# *GFAP* canonical transcript may not be suitable for the diagnosis of adult-onset Alexander disease

**DOI:** 10.1186/s40478-018-0616-z

**Published:** 2018-10-24

**Authors:** Filippo Pinto e Vairo, Nicole Bertsch, Eric W. Klee, Ralitza H. Gavrilova

**Affiliations:** 1 Center for Individualized Medicine, Mayo Clinic, Harwick 3, 200 First Street SW, Rochester, MN 55905 USA; 2 Department of Health Sciences Research, Mayo Clinic, Rochester, MN USA; 3 Department of Clinical Genomics, Mayo Clinic, Rochester, MN USA; 4 Department of Biomedical Informatics, Mayo Clinic, Rochester, MN USA; 5 Department of Neurology, Mayo Clinic, Rochester, MN USA

**Keywords:** Adult-onset Alexander disease, Sanger sequencing, Alternative transcripts, GFAP

**Fig. 1:**
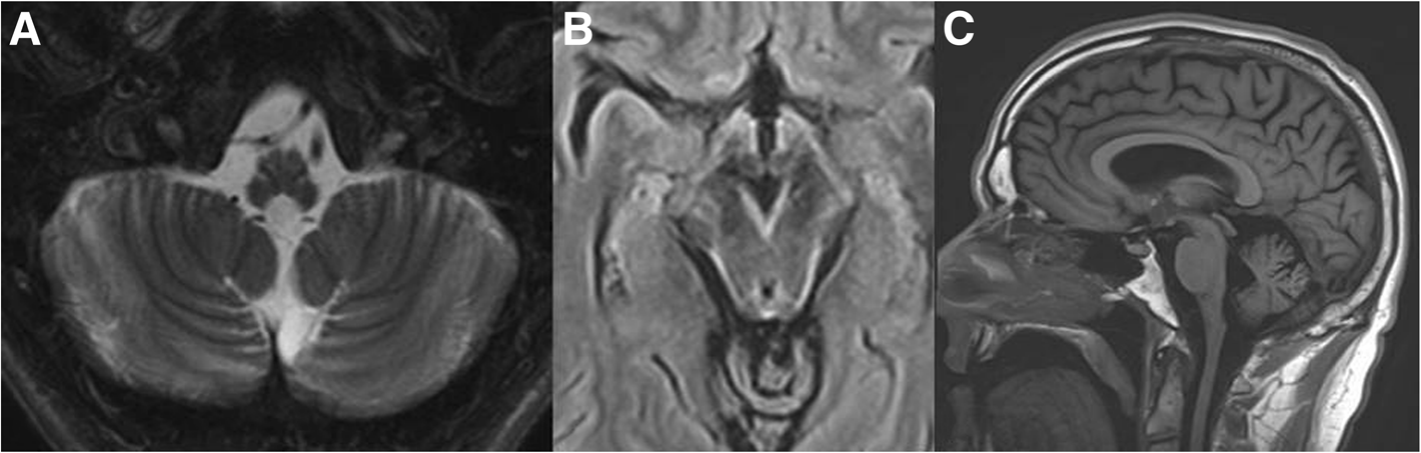
**a** Severe atrophic changes of the medulla including the inferior olivary nuclei. Moderate cerebellar atrophy. **b** Linear symmetric abnormal T2 signal predominantly along the anteriolateral aspect of the medulla and a thin zone of abnormal T2 signal about the periphery of the midbrain. **c** Sagittal view showing atrophy of the brainstem and upper cervical cord. No changes were noted in the subcortical regions

To the editor,

Alexander disease (AD) is an autosomal dominant progressive leukoencephalopathy caused by heterozygous mutations in the glial fibrillary acidic protein (*GFAP*) gene. Onset of symptoms can range from infancy to adulthood, with the infantile form being most common and the adult onset form accounting for one third of cases [4]. A clinical diagnosis is typically confirmed via sequencing of the *GFAP* gene, as 98% of the individuals have detectable single nucleotide or small indels pathogenic variants*.* Here we highlight the emerging importance of testing an exon only expressed in an alternative *GFAP* transcript, which has recently been shown to harbor pathogenic variation found in later-onset patient presentations. A brief survey of clinical testing laboratories reveals that not all include this exon in the reportable range of *GFAP* testing and consequently fail to reveal the correct genetic diagnosis. This point is highlighted in a 43-year-old male patient was referred to Clinical Genomics for evaluation of hereditary hemorrhagic telangiectasia (HTT) based on positive family history and a variant of uncertain significance (VUS) in *ACVRL1* (c.106T > C; p.Cys36Arg). In addition, he presented with a family history of an unidentified leukodystrophy in mother and maternal uncle, and a potential AD diagnosis based on the characteristic distribution of Rosenthal fibers in uncle’s autopsy and mother’s neurological symptoms. His mother was 67 years old and had history for about four years of progressive worsening gait, spasticity, and slurred speech. MRI revealed leukodystrophy. The patient was asymptomatic with a normal neurological examination (no gait abnormality, abnormal posture, dysarthria, or other symptoms related to AD) but a brain MRI demonstrated findings previously described in adult-onset AD (Fig. 1) [3]. *GFAP* sequencing (NM_002055.3 transcript) done at a CLIA laboratory revealed no pathogenic variants. Additional genetic evaluation for adult-onset leukoencephalopathies was also negative. Subsequent research whole exome sequencing (WES) detected a pathogenic variant in a deep intronic region of the canonical transcript (c.1171 + 472G > A) as well as in exon 7 of an alternative transcript (NM_001131019.2(GFAP):c.1289G > A; p.(Arg430His)). This variant has been previously reported in two affected individuals with clinical and MRI findings of AD and a variant in *HDAC6*, a possible modifier gene [2]. Noteworthy, we did not find variants in *HDAC6* or other known modifiers in our patient. The *GFAP* variant is present in 2 of 245,904 alleles in gnomAD [1]. Since the disease is thought to be fully penetrant, there may be two other presently asymptomatic/oligosymptomatic individuals who remain undiagnosed. Therefore, when the diagnosis is clinically suspected, alternative molecular analysis may be warranted to detect changes in all relevant transcripts. Moreover, our patient is the third reported case with the same causative variant in an alternative *GFAP* exon, making this variant a recurrent cause of adult-onset AD. We believe that the available clinical genetic testing for AD should be revisited and include this alternate exon.
